# Microbial solvent formation revisited by comparative genome analysis

**DOI:** 10.1186/s13068-017-0742-z

**Published:** 2017-03-09

**Authors:** Anja Poehlein, José David Montoya Solano, Stefanie K. Flitsch, Preben Krabben, Klaus Winzer, Sharon J. Reid, David T. Jones, Edward Green, Nigel P. Minton, Rolf Daniel, Peter Dürre

**Affiliations:** 1 Genomic and Applied Microbiology and Göttingen Genomics Laboratory, Georg-August University Göttingen, Grisebachstr. 8, 37077 Göttingen, Germany; 2 Institut für Mikrobiologie und Biotechnologie, Universität Ulm, Albert-Einstein-Allee 11, 89081 Ulm, Germany; 3 Green Biologics Ltd., 45A Western Avenue, Milton Park, Abingdon, Oxfordshire OX14 4RU UK; 4 Clostridia Research Group, BBSRC/EPSRC Synthetic Biology Research Centre (SBRC), School of Life Sciences, University of Nottingham, Nottingham, NG7 2RD UK; 5 Department of Molecular and Cell Biology, University of Cape Town, Rondebosch, Cape Town, 7701 South Africa; 6 Department of Microbiology and Immunology, University of Otago, Dunedin, 9010 New Zealand; 7 CHAIN Biotechnology Ltd., Imperial College Incubator, Level 1 Bessemer Building, Imperial College London, London, SW7 2AZ UK

**Keywords:** Acetone, Butanol, *Clostridium acetobutylicum*, *C. beijerinckii*, *C. saccharobutylicum*, *C. saccharoperbutylacetonicum*, Phylogeny, Solvents

## Abstract

**Background:**

Microbial formation of acetone, isopropanol, and butanol is largely restricted to bacteria belonging to the genus *Clostridium*. This ability has been industrially exploited over the last 100 years. The solvents are important feedstocks for the chemical and biofuel industry. However, biological synthesis suffers from high substrate costs and competition from chemical synthesis supported by the low price of crude oil. To render the biotechnological production economically viable again, improvements in microbial and fermentation performance are necessary. However, no comprehensive comparisons of respective species and strains used and their specific abilities exist today.

**Results:**

The genomes of a total 30 saccharolytic *Clostridium* strains, representative of the species *Clostridium acetobutylicum*, *C. aurantibutyricum*, *C. beijerinckii*, *C. diolis*, *C. felsineum*, *C. pasteurianum*, *C. puniceum*, *C. roseum*, *C. saccharobutylicum*, and *C. saccharoperbutylacetonicum*, have been determined; 10 of them completely, and compared to 14 published genomes of other solvent-forming clostridia. Two major groups could be differentiated and several misclassified species were detected.

**Conclusions:**

Our findings represent a comprehensive study of phylogeny and taxonomy of clostridial solvent producers that highlights differences in energy conservation mechanisms and substrate utilization between strains, and allow for the first time a direct comparison of sequentially selected industrial strains at the genetic level. Detailed data mining is now possible, supporting the identification of new engineering targets for improved solvent production.

**Electronic supplementary material:**

The online version of this article (doi:10.1186/s13068-017-0742-z) contains supplementary material, which is available to authorized users.

## Background

Acetone and butanol are important solvents that are used to manufacture adhesives, cosmetics, lacquers, paints, plastics, pharmaceuticals, and polymers in combined chemical markets worth more than $6 billion [1]. Today, most of this market demand is met with solvents derived from oil. During the first part of the last century, the production of these solvents via the acetone–butanol–ethanol (ABE) fermentation process served as the major source of industrial solvents. Solvent-producing clostridia became a focus of interest during the early 1900s, due to their potential for the commercial production of solvents. Initial studies were centered on production of butanol for the manufacture of synthetic rubber. With the advent of WW1, emphasis rapidly shifted to the production of acetone that was needed in large volumes for the production of munitions. In 1915, Charles (later Chaim) Weizmann from the University of Manchester was granted his famous patent for the production of acetone and butanol using an anaerobic bacterium [2]. This organism was later named *Clostridium acetobutylicum* [3]. During WW1, the production of acetone on industrial scale was undertaken in the UK, France, Canada, and the USA and played a vital role in munitions’ production for the Allies. Weizmann’s contribution was recognized by the British Government and played a part in the Balfour declaration in 1917, providing the initial nucleus for founding the state of Israel in 1948, with Weizmann becoming the countries first president [4, 5].

After the war, the need for large volumes of acetone fell away and butanol production became the main commercial focus. The Weizmann process and patent were acquired by the Commercial Solvent Corporation (CSC) in the US and the company remained the sole producer of solvents until the patent expired in 1930. During the 1930s, three other US chemical companies established their own, independent, industrial ABE processes and ABE plants were also established in Cuba, Puerto Rico, and South Africa. Beginning in the 1920s, Japan also embarked a major program for the production of butanol as an aviation fuel supplement. This government program eventuated in the building of numerous ABE plants in Japan and Taiwan prior to and during WW2 [6]. The Japanese program was initially based on a derivative of the Weizmann strain before the isolation and development of Japanese solvent-producing strains. None of these early strains appear to have survived, but some successful industrial strains designated *C. saccharoperbutylacetonicum*, from the post war period, were lodged with international strain collections.

During the 1930s, the expanding sugar industry resulted in a world-wide glut in molasses and an overproduction of sugar cane juice. This resulted in the fermentation industry switching to this abundant, much cheaper substrate. The *C. acetobutylicum* strain patented by Weizmann and its various derivatives that were developed to produce solvent from corn and other starch-based substrates proved to be unsuitable for use on molasses and similar sugar-based substrates. From the 1930s, all four of the US companies utilized molasses as the substrate for the ABE fermentation. This involved each of the US companies in the isolation, selection, and development of their own closely guarded, in house, solvent-producing strains for use on molasses. Some of these strains were also able to reduce acetone further to isopropanol. Many of these were patented under a multiplicity of different names [5]. Unfortunately, the only examples of this new generation of industrial saccharolytic strains to have survived are those developed and patented by CSC along with some later strains developed by McCoy, who had worked as a consultant for CSC. These included strains utilized in the Puerto Rico process. As a joint venture, CSC established a new molasses-based ABE plant in the UK in 1935 utilizing the new generation of CSC industrial stains. The National Chemical Products (NCP) plant established in South Africa originally utilized a French derivative of the Weizmann strain using corn as the substrate. During WW2, the NCP plant in South Africa was converted to using molasses as the substrate.

The NCP industrial strain collection is the most complete collection of ABE bacteria and based on strains originally supplied by CSC, from the US, during 1944 and 1945 with further strains supplied by Commercial Solvents-Great Britain (CS-GB) in 1951. The main CSC industrial strains were patented under the names of *C. saccharo*-*acetobutylicum*, *C. granulobacter acetobutylicum*, and *C. saccharo*-*butyl*-*acetonicum*-*liquifaciens* [7]. A strain of *C*. *saccharo*-*acetobutylicum* is now known as *C. beijerinckii* NRRL B-591/NCIMB 8052. The later *C. granulobacter acetobutylicum* strains were transferred to NCP and are now classified as NCP *C. beijerinckii* strains. The *C. saccharo*-*butyl*-*acetonicum*-*liquifaciens* strains were also transferred to NCP and are now classified as *C. saccharobutylicum*.

The ABE fermentation flourished in the US, the UK, and Japan until the 1950s, when solvents manufactured from cheap crude oil made the ABE fermentation process increasingly uneconomic. The ABE plant in the UK ceased operation in 1959. The ABE plants in Japan closed in the early 1960s. The last ABE plant in the US operated by Publicker Industries ceased operation in 1977. South Africa operated an ABE plant until 1983, while China continued to maintain several plants and, in 2006, established several new ones. However, these soon became uneconomic due to decreasing oil price and most were closed by 2009.

More recently, Green Biologics has applied modern microbiology and advanced engineering to the conventional ABE fermentation process. The company has constructed a renewable chemicals facility in Little Falls, Minnesota by retrofitting a 21 million gallon-per year ethanol plant with their advanced *Clostridium* fermentation technology to produce bio-based butanol and acetone for chemical applications. Production is expected to ramp up to full capacity during 2017.

Better understanding and intimate knowledge of genome sequence from industrial strains, used commercially over 70 years, will support efforts to engineer and develop superior microbes for solvent production. There is a need to develop robust and highly productive strains that can utilize low cost and sustainable renewable feedstocks and make a significant contribution toward a more economically viable and environmentally friendly fermentation route for commodity chemical and biofuel production.

## Results

### Phylogeny and taxonomy

**Fig. 1:**
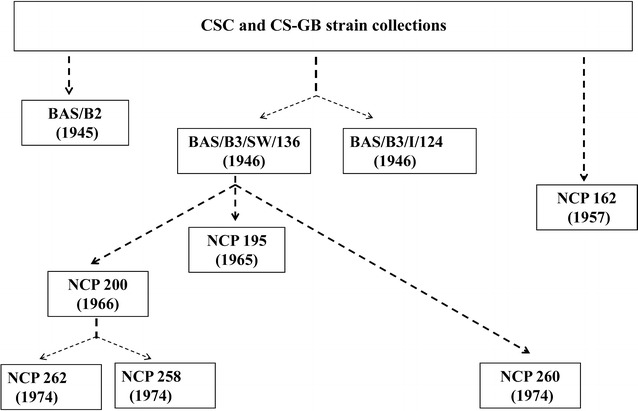
Historical development of industrial ABE strains: only sequenced strains are indicated. Data stem from Jones [7]

**Fig. 2:**
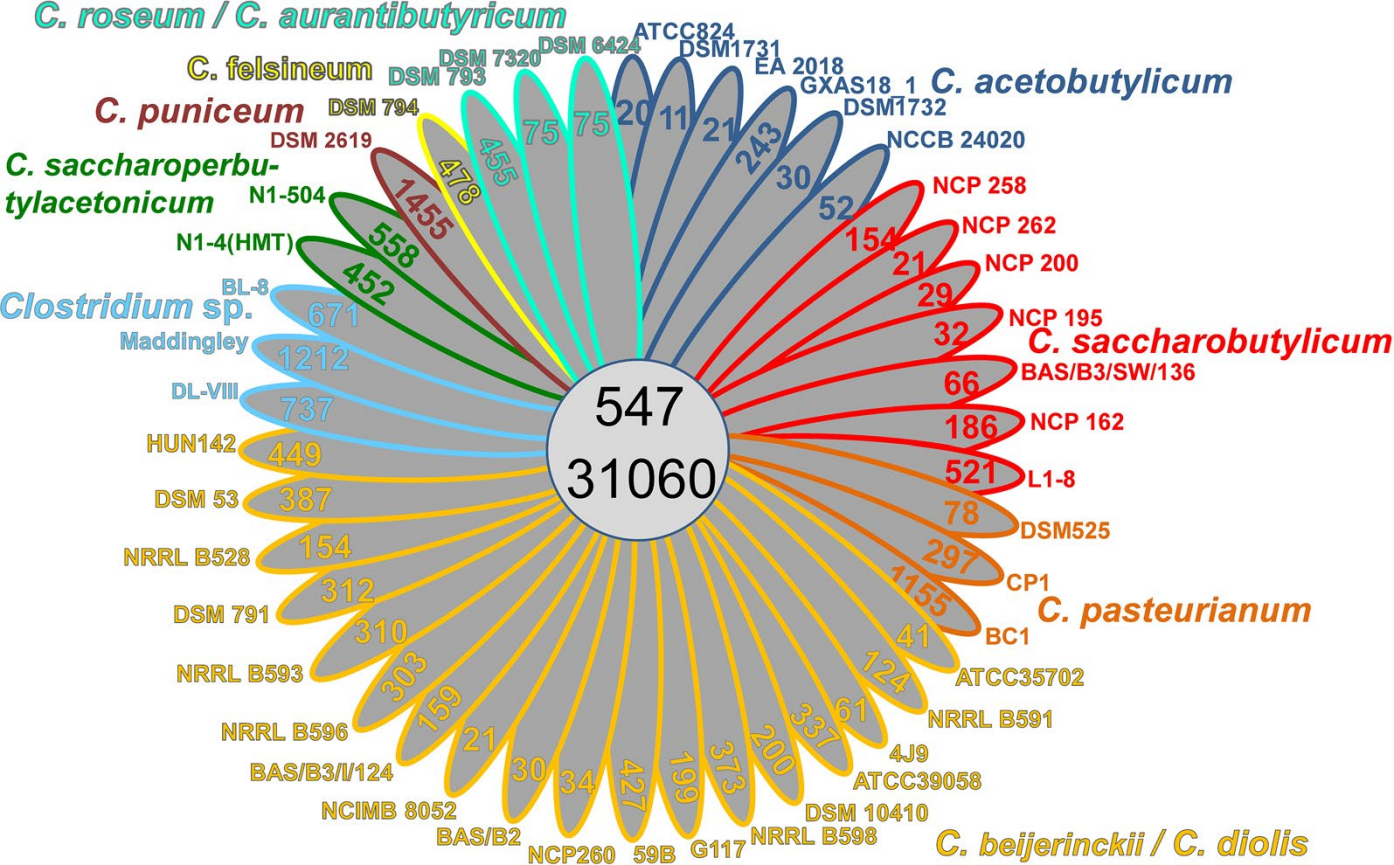
Core/Pan genome analysis of 44 clostridial genomes: a simplified Venn diagram showing the core and the pan genome of all 44 solventogenic clostridia. The number of genome-specific OGs is depicted in the respective ellipse. Ortholog detection was done with blastp and the Proteinortho software [8] with a similarity cutoff of 50% and an *E* value of 1e−10

Until recently, only the sequences of some *C. acetobutylicum*, *C. beijerinckii* strains, and *C. diolis* were publicly available, but many other species such as *C. aurantibutyricum*, *C. felsineum*, *C. pasteurianum*, *C. puniceum*, *C. roseum*, *C. saccharobutylicum*, and *C. saccharoperbutylacetonicum* are able to perform ABE fermentation. Genomes from all these species, including all type strains, were sequenced. Genomes of *C. saccharobutylicum* strains BAS/B3/SW/136, NCP 195, NCP 200, NCP 258, DSM 13864, of *C. saccharoperbutylacetonicum* strains N1-4 (HMT), N1-504, of *C. pasteurianum* DSM 525, and of *C. beijerinckii* BAS/B3/I/124 and 59B were closed, all other genomes are draft form (Table 1). The historical development of the sequenced industrial strains is depicted in Fig. 1. Genome sizes vary between 4.099 Mb (*C. acetobutylicum* NCCB 24020) and 6.666 Mb [*C. saccharoperbutylacetonicum* N1-4 (HMT)]. The latter is the largest genome within the solventogenic clostridia. We found the lowest number of genes (around 4000) in the genomes of the *C. acetobutylicum* species and the highest number (5937) in *C. saccharoperbutylacetonicum* N1-4 (HMT). To correlate metabolic potential with strain phylogeny, we compared our newly derived genome sequences with those that are publicly available. A whole genome comparison based on protein-encoding genes revealed a core genome shared by all 44 strains of 547 orthologous groups (OGs) and a pan genome of 31,060 OGs (Fig. 2). There was a broad range of genome-specific OGs (singletons) varying between 11 and 737, which is, with three exceptions, smaller than the core genome of all 44 strains studied. Three genomes, namely *C. pasteurianum* BC1, *Clostridium* sp. Maddingley MBC34-24, and *C. puniceum* DSM 2619 encoded 1155, 1212 and 1455 singletons, respectively, which is 2–3 times higher than the core genome of all analyzed strains.

**Table 1:** General features of newly sequenced strains

Organism	Type strain/industrial strain	Size (bp)	Scaffolds	GC content (%)	Coding percentage (%)	CDS	Genes	rRNA	tRNA	Coverage illumina/454	Sequencing platform	Read length illumina (bp)
*Clostridium acetobutylicum* DSM 1732	Industrial strain	4,091,215	55	30.69	86.96	3871	3934	2	60	251	Genome Analyzer IIx	2 × 112
*Clostridium acetobutylicum* NCCB 24020		4,098,731	20	30.71	87.17	3883	3970	8	78	157	MiSeq	2 × 300
*Clostridium aurantibutyricum* DSM 793T	Type strain	4,922,827	221	29.87	86.04	4492	4572	10	70	128	MiSeq	2 × 300
*Clostridium beijerinckii* 4J9		5,888,124	162	29.60	80.34	5200	5271	7	63	145	Genome Analyzer IIx	2 × 112
*Clostridium beijerinckii* ATCC 39058		5,953,339	302	29.57	80.32	5284	5297	1	11	233	HiSeq2000	2 × 100
*Clostridium beijerinckii* BAS/B2	Industrial strain	5,982,920	245	29.61	81.03	5235	5294	10	48	307	HiSeq2000	2 × 51
*Clostridium beijerinckii* BAS/B3/I/124	Industrial strain	6,123,550	1	29.87	80.56	5310	5435	43	77	123/15	Genome Analyzer IIx/454-GS FLX	2 × 112
*Clostridium beijerinckii* DSM 53		5,773,247	346	29.54	80.15	5057	5126	12	56	225	HiSeq2000	2 × 51
*Clostridium beijerinckii* DSM 791T	Type strain	5,781,472	264	29.66	79.99	5081	5184	16	86	131	MiSeq	2 × 300
*Clostridium beijerinckii* NCP 260	Industrial strain	5,968,330	242	29.61	81.03	5223	5286	8	54	228	HiSeq2000	2 × 51
*Clostridium beijerinckii* NRRL B-528		6,255,488	233	29.64	79.60	5554	5661	17	89	68	MiSeq	2 × 301
*Clostridium beijerinckii* NRRL B-591	Industrial strain	5,874,824	358	29.58	79.97	5162	5176	2	11	264	HiSeq2000	2 × 100
*Clostridium beijerinckii* NRRL B-593		6,156,662	305	29.57	79.74	5469	5525	7	49	192	HiSeq2000	2 × 100
*Clostridium beijerinckii* NRRL B-596		6,220,133	393	29.59	80.80	5531	5547	2	13	152	HiSeq2000	2 × 100
*Clostridium beijerinckii* 59B		6,485,394	1	30.00	78.79	5522	5670	49	93	800	HiSeq2000/454-GS FLX	2 × 51
*Clostridium felsineum* DSM 794T	Type strain	5,178,654	105	29.92	86.80	4745	4831	9	77	82	MiSeq	2 × 300
*Clostridium pasteurianum* DSM 525T	Type strain	4,352,101	1	29.94	82.54	3988	4099	30	81	70/17	MiSeq/454-GS FLX	2 × 51
*Clostridium puniceum* DSM 2619T	Type strain	6,082,167	245	28.61	80.07	5305	5373	13	54	103	MiSeq	2 × 300
*Clostridium roseum* DSM 6424		4,944,863	262	29.75	86.48	4510	4529	1	18	152	HiSeq2000	2 × 100
*Clostridium roseum* DSM 7320T	Type strain	5,067,725	124	29.80	87.35	4607	4687	8	72	84	MiSeq	2 × 300
*Clostridium saccharobutylicum* BAS/B3/SW/136	Industrial strain	5,108,304	1	28.67	78.90	4383	4521	37	93	123	Genome Analyzer IIx/454-GS FLX	2 × 112
*Clostridium saccharobutylicum* L1-8		5,173,344	16	28.60	78.97	4487	4596	28	80	157/10	HiSeq2000/454-GS FLX	2 × 51
*Clostridium saccharobutylicum* NCP 162	Industrial strain	4,900,327	142	28.46	78.95	4320	4381	8	52	198	Genome Analyzer IIx	2 × 112
*Clostridium saccharobutylicum* NCP 195	Industrial strain	5,108,176	1	28.66	78.81	4377	4514	37	89	198	Genome Analyzer IIx/454-GS FLX	2 × 112
*Clostridium saccharobutylicum* NCP 200	Industrial strain	5,108,287	1	28.67	78.86	4380	4518	37	91	92	Genome Analyzer IIx/454-GS FLX	2 × 112
*Clostridium saccharobutylicum* NCP 258	Industrial strain	4,950,933	1	28.66	78.67	4296	4436	37	85	111	HiSeq2000/454-GS FLX	2 × 51
*Clostridium saccharobutylicum* DSM 13864T	Type strain/industrial strain	5,107,814	1	28.66	79.15	4469	4593	37	85	100/29	HiSeq1000/454-GS FLX	2 × 32
*Clostridium saccharoperbutylacetonicum* N1-4 (HMT)T	Type strain/industrial strain	6,666,445	2	29.54	82.91	5821	5937	35	70	43/15	Genome Analyzer IIx/454-GS FLX	2 × 112
*Clostridium saccharoperbutylacetonicum* N1-504		6,219,394	2	29.55	83.02	5518	5622	34	60	113	HiSeq2000	2 × 50
Clostridium sp. BL-8		6,045,940	231	29.89	81.68	5450	5466	3	13	176	HiSeq2000	2 × 100

**Fig. 3:**
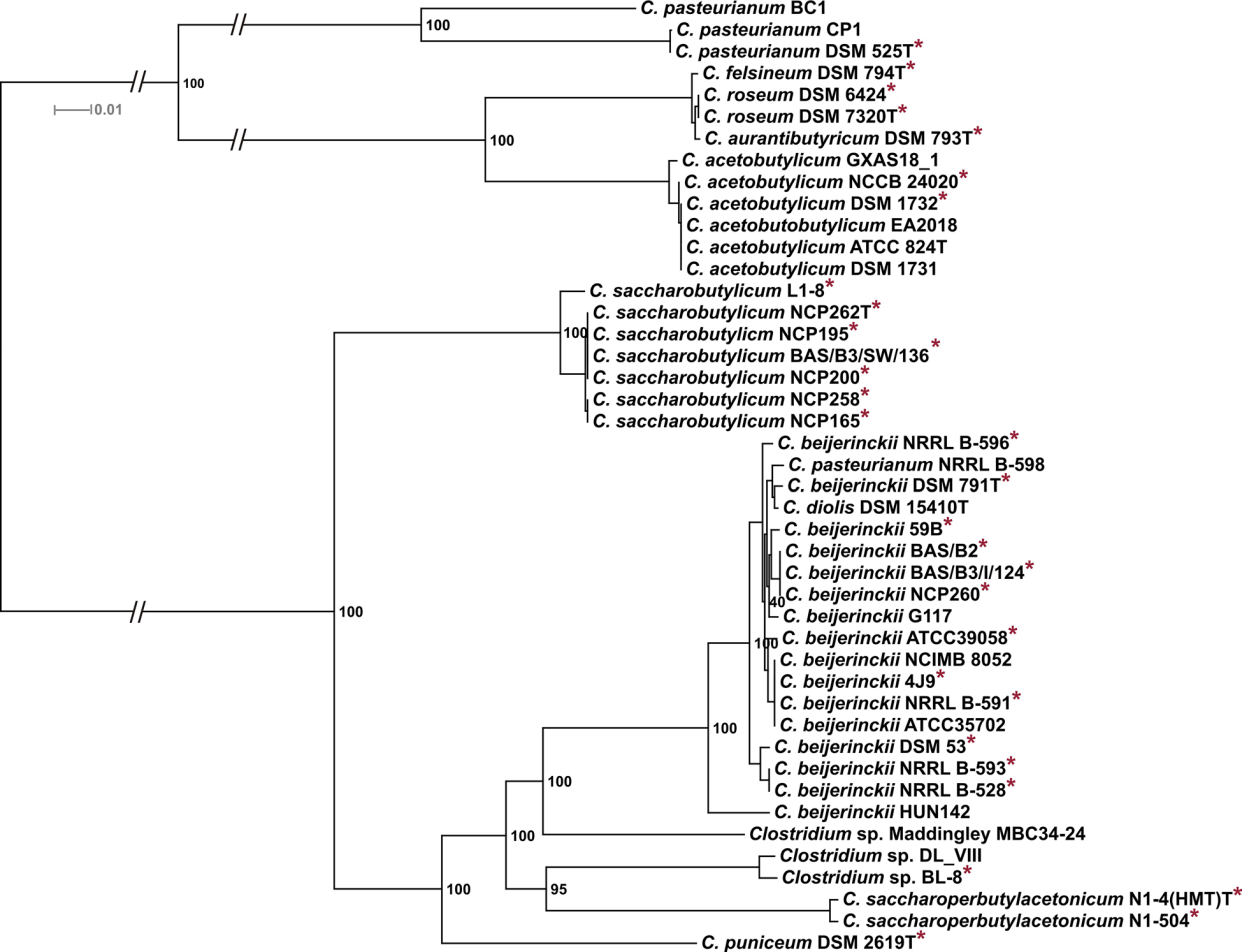
MLSA tree of 44 sequenced solventogenic clostridia: a maximum likelihood tree of 44 solventogenic clostridial genomes was inferred with 500 bootstraps with RAxML [9] and visualized with Dendroscope [10]. Genomes sequenced within this study were marked with a *red asterisk* and type strains marked with a T

**Fig. 4:**
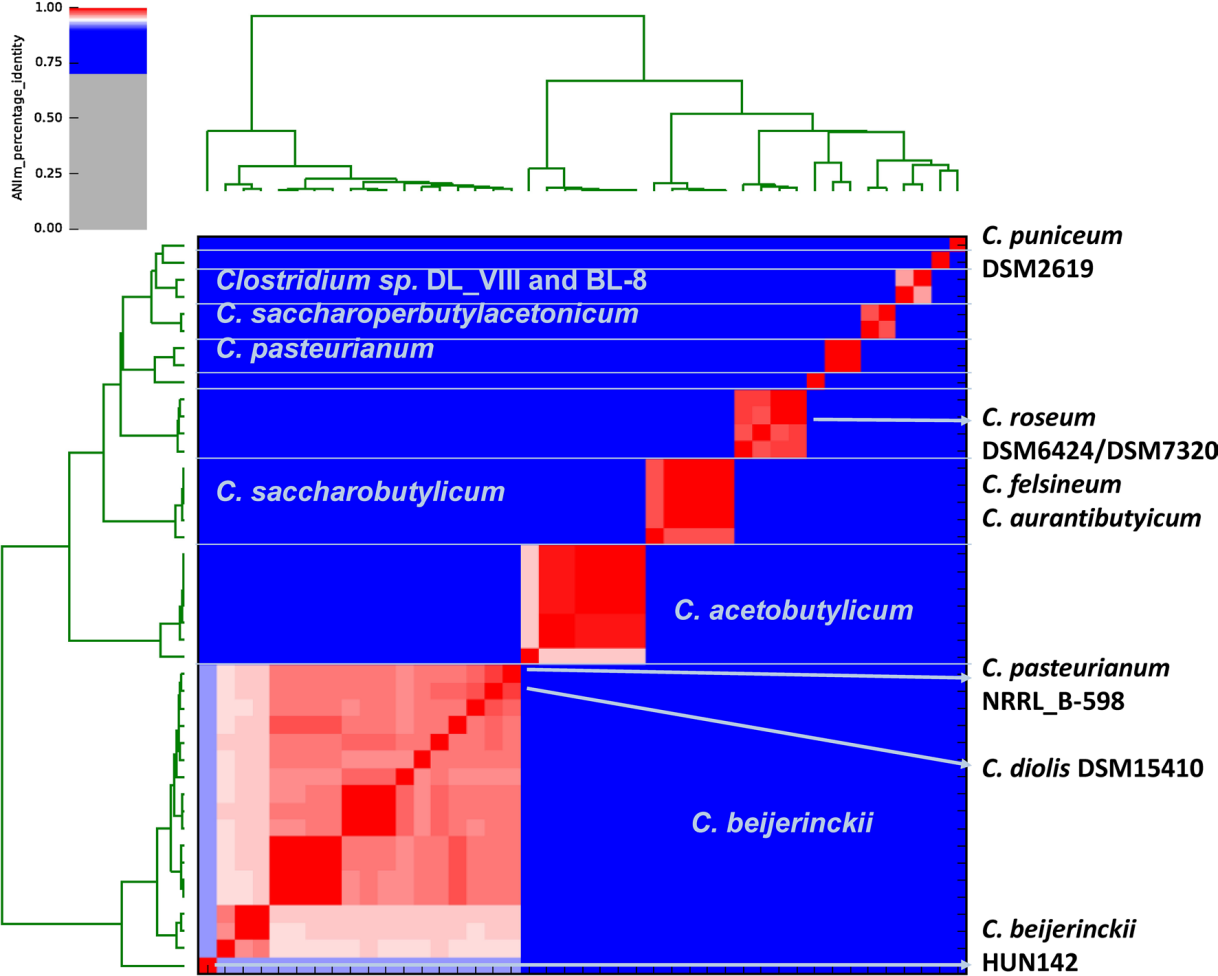
Average nucleotide identity analysis of the 44 sequenced strains: ANI analysis based on MUMmer alignment of the genome sequences was performed and visualized using PYANI [13]

The phylogeny of the strains was analyzed by multi-locus sequence analysis (MLSA) based on the detected core genome (Fig. 3). The phylogenic tree yielded two main clades (I and II) with several subclades. The first comprises a *C. acetobutylicum*, a *C. roseum*/*C. aurantibutyricum*/*C. felsineum*, and a *C. pasteurianum* subclade, whereas *C. pasteurianum* BC1 branches outside the last-mentioned subclade. The second main clade consists of a *C. saccharobutylicum*, *C. beijerinckii* subcluster, which includes *C. diolis* DSM 15410 and *C. pasteurianum* NRRL B-598, a *C. saccharoperbutylacetonicum* subclade, and a subcluster consisting of *Clostridium* sp. DL_VIII and BL-8. The genomes of *Clostridium* sp. Maddingley MBC34-24 and *C. puniceum* DSM 2619 branch outside the other subclades of main clade II. This result correlates with the core/pan genome analysis, as these strains, together with *C. pasteurianum* BC1, represent the strains with the highest number of singletons, indicating that these strains are distantly related to the other analyzed strains or species. Whilst MLSA can provide insight into the phylogenetic relationship of organisms, for taxonomic studies, other methods, such as Average Nucleotide Identity (ANI) analysis [11], a suitable in silico alternative for DNA–DNA hybridization [12], are required. We performed an ANI analysis based on MUMmer alignment (ANIm) of the 44 genomes to define species and their complexes (Fig. 4). We identified a large *C. beijerinckii* species complex consisting of 17 strains including *C. diolis* DSM 15410 and *C. pasteurianum* NRRL B-598 having ANIm values between 96 and 100% (Additional file 1: Table S1) compared to all other *C. beijerinckii* strains, which is clearly above the species threshold. The second species complex comprises all *C. saccharobutylicum* strains and our analysis demonstrates that strain L1-8 is a different subtype compared to the other strains. Our analysis also revealed that all *C. acetobutylicum* strains are very closely related (ANIm values of 100%), with the exception of strain GXAS18_1 (ANIm of 98%). In this strain, the contigs representing the *sol* operon are missing in the publicly available genome sequence. We identified a quite diverse species complex consisting of *C. roseum* DSM 7320 and DSM 6424, *C. aurantibutyricum* DSM 793, and *C. felsineum* DSM 794, but ANIm values between 98 and 100% clearly showed that these organisms represent one species and, based on whole genome sequence comparison, these organisms have to be reclassified. Based on ANIm analysis, *Clostridium* sp. BL-8 and DL_VIII belong to the same species, but not to any of the described species able to perform ABE fermentation. Our analysis also showed that *C. beijerinckii* HUN142 and *C. pasteurianum* BC1 do not belong to the *C. beijerinckii* and the *C. pasteurianum* species complex, respectively and that *Clostridium* sp. Maddingley MBC34-24 and *C. puniceum* DSM 2619, respectively, have no close relative and do not belong to any of the described ABE species.

### Plasmids

Plasmids have been found in 13 of the 44 analyzed ABE strains. The megaplasmid pSOL1 of *C. acetobutylicum* ATCC824 with a size of 192,000 bp is indispensable for solvent formation [14]. The strains *C. acetobutylicum* DSM1731, DSM1732, EA2018, and NCCB24020 carry similar megaplasmids, which also contain the *sol*–*adc* gene cluster. In addition, strain DSM1731 contains an 11,100-bp plasmid with an unknown role in clostridial physiology [15]. *C. saccharoperbutylacetonicum* N1-4 (HMT) contains a megaplasmid of 136,188 bp without genes apparently related to solvent formation. Strain N1-504 carries the 2936-bp plasmid pNAK1, which is identical to pCS86 from *C. acetobutylicum* 86 that has been used in the past for shuttle vector construction [16]. *C. beijerinckii* strains HUN142 and NRRL B-593 carry mostly cryptic plasmids ranging from <2000 to 65,000 bp. An exception is a 65,000-bp plasmid of *C. beijerinckii* HUN142, which contains genes for defense (lantibiotics, proteases), antibiotic resistance, and quorum sensing. All strains of *C. aurantibutyricum* and *C. roseum* carry plasmids ranging from 31,015 to 55,559 bp. The misclassified *C. pasteurianum* BC1 strain also contains a plasmid with a size of 53,393 bp, and *C. felsineum* carries a megaplasmid (339,775 bp), containing genes involved in spore germination. A detailed analysis on presence and sizes of plasmids is presented in Additional file 2: Table S2.

### Genes required for acidogenesis and solventogenesis

**Fig. 5:**
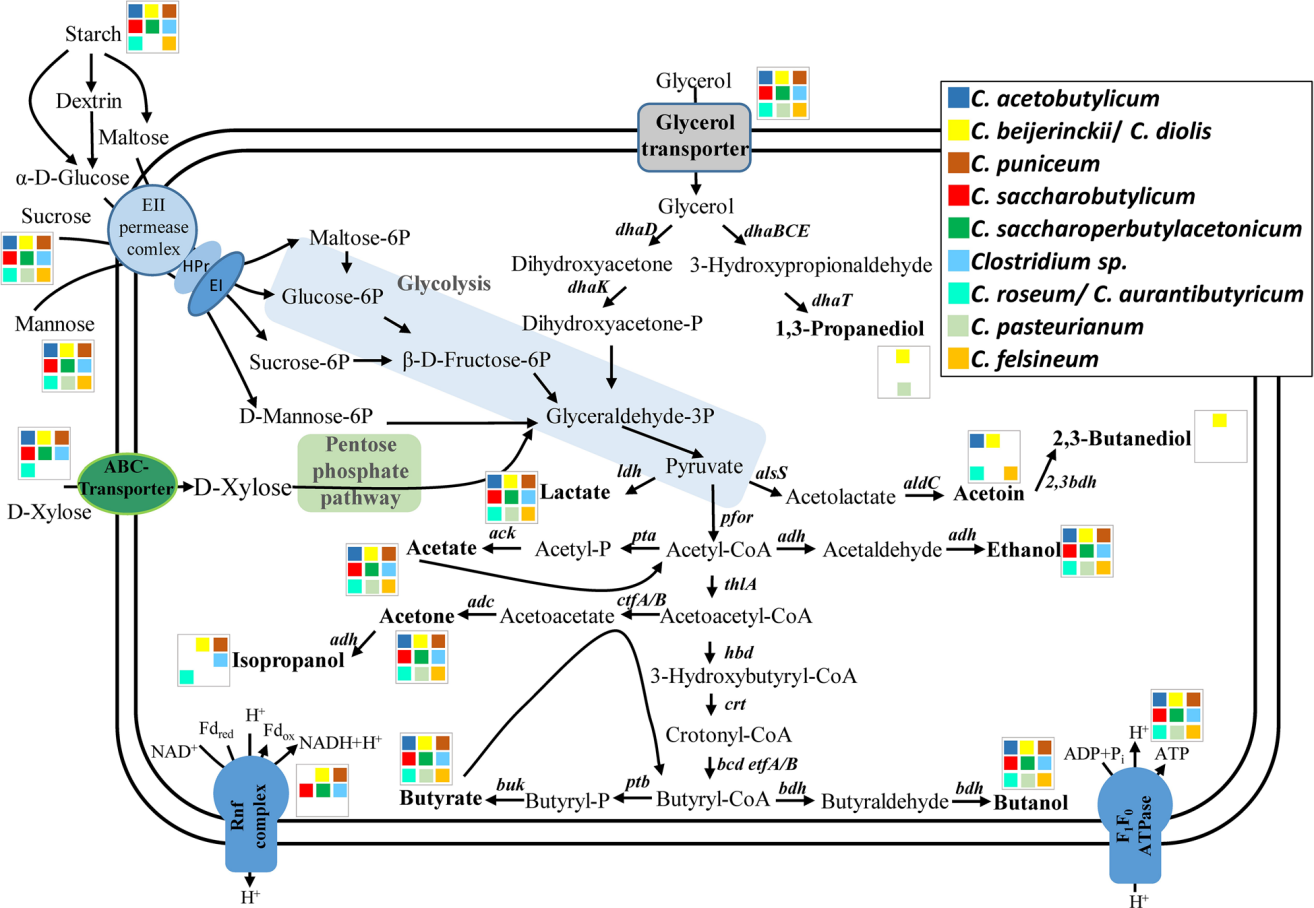
Central metabolism of solventogenic clostridia: *Color codes* indicate the presence or absence of specific enzymes in the various species of solventogenic clostridia. Position and colors are always conserved from left to right: *First row C. acetobutylicum*, *C. beijerinckii/C. diolis*, *C. puniceum*; *second row C. saccharobutylicum*, *C. saccharoperbutylacetonicum*, *Clostridium* sp.; *third row C. roseum/C. aurantibutyricum*, *C. pasteurianum*, *C. felsineum*. *Blanks* (*white*) indicate absence of respective enzymes

The predominant acids formed are acetate and butyrate. Both are produced from their respective coenzyme A derivatives via transphosphorylases and kinases (Fig. 5). Genes for phosphotransacetylase and acetate kinase (*pta* and *ack*, respectively) as well as phosphotransbutyrylase and butyrate kinase (*ptb* and *buk*, respectively) are organized in bi-cistronic operons in all strains analyzed. Butyrate formation starts by formation of acetoacetyl-CoA from two acetyl-CoA (catalyzed by thiolase). The following steps, conversion of acetoacetyl-CoA to butyryl-CoA, are catalyzed by enzymes whose genes are clustered in all of the strains analyzed. The order of genes in this *bcs* (butyryl-CoA synthesis) cluster [17] is also conserved as *crt*–*bcd*–*etfB*–*etfA*–*hbd*. Analysis of putative terminators with EMBOSS and DNAsis revealed the expected terminators directly upstream of *crt* and downstream of *hbd*. Curiously, a hairpin structure without T-rich region was found between the genes *etfA* and *hbd* in all analyzed phylogenetic clusters. It may represent a former junction formed when the *bcs* operon was integrated during evolution or might be involved in independent regulation of the *bcs* operon under certain growth conditions. Lactate is only formed under specific conditions [18]. All strains analyzed carry a lactate dehydrogenase gene. A previous report, comparing only the genomes of the two strains *C. acetobutylicum* ATCC 824 and *C. beijerinckii* NCIMB 8052, indicated the presence of a pyruvate decarboxylase gene only in *C. acetobutylicum* and of genes encoding a trimeric bifurcating hydrogenase only in *C. beijerinckii* [19]. We could confirm that a *pdc* gene is indeed only present in the *C. acetobutylicum*, *C. aurantibutyricum/C. felsineum/C. roseum*, and *C. pasteurianum* clade. With respect to the bifurcating hydrogenase, the result is not that unambiguous. The *C. acetobutylicum*, *C. aurantibutyricum/C. felsineum/C. roseum*, and *C. pasteurianum* clade lacks all three genes, but the *C. saccharobutylicum* strains and *C. puniceum* lack only one of these genes.

**Fig. 6:**
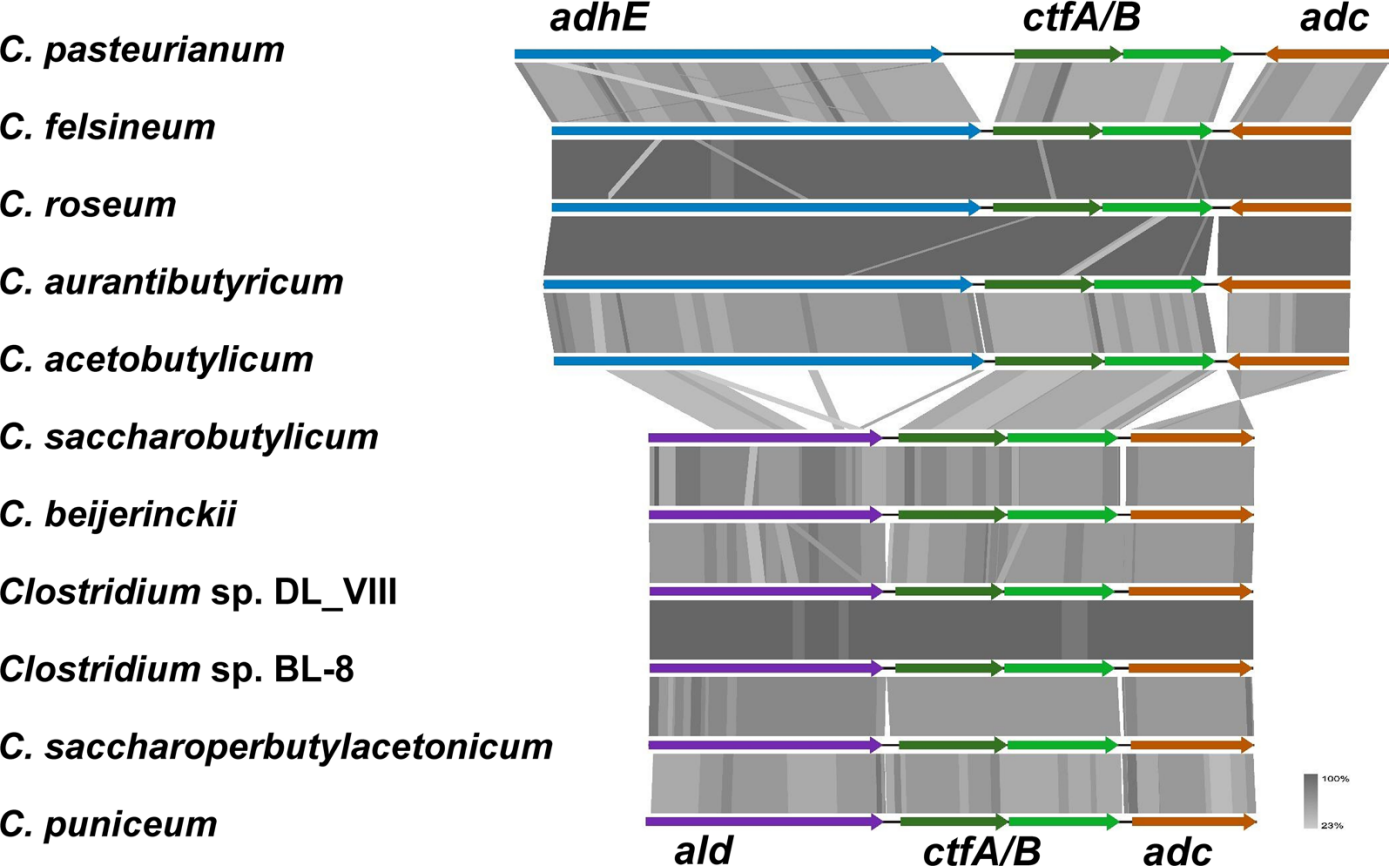
Structure of the *sol* operon: structure of the *sol* operon based on Tblastx comparison of representative members of the different subclades. An *E* value cutoff of 1e−10 was used and visualization were done with the program Easyfig [21]

**Fig. 7:**
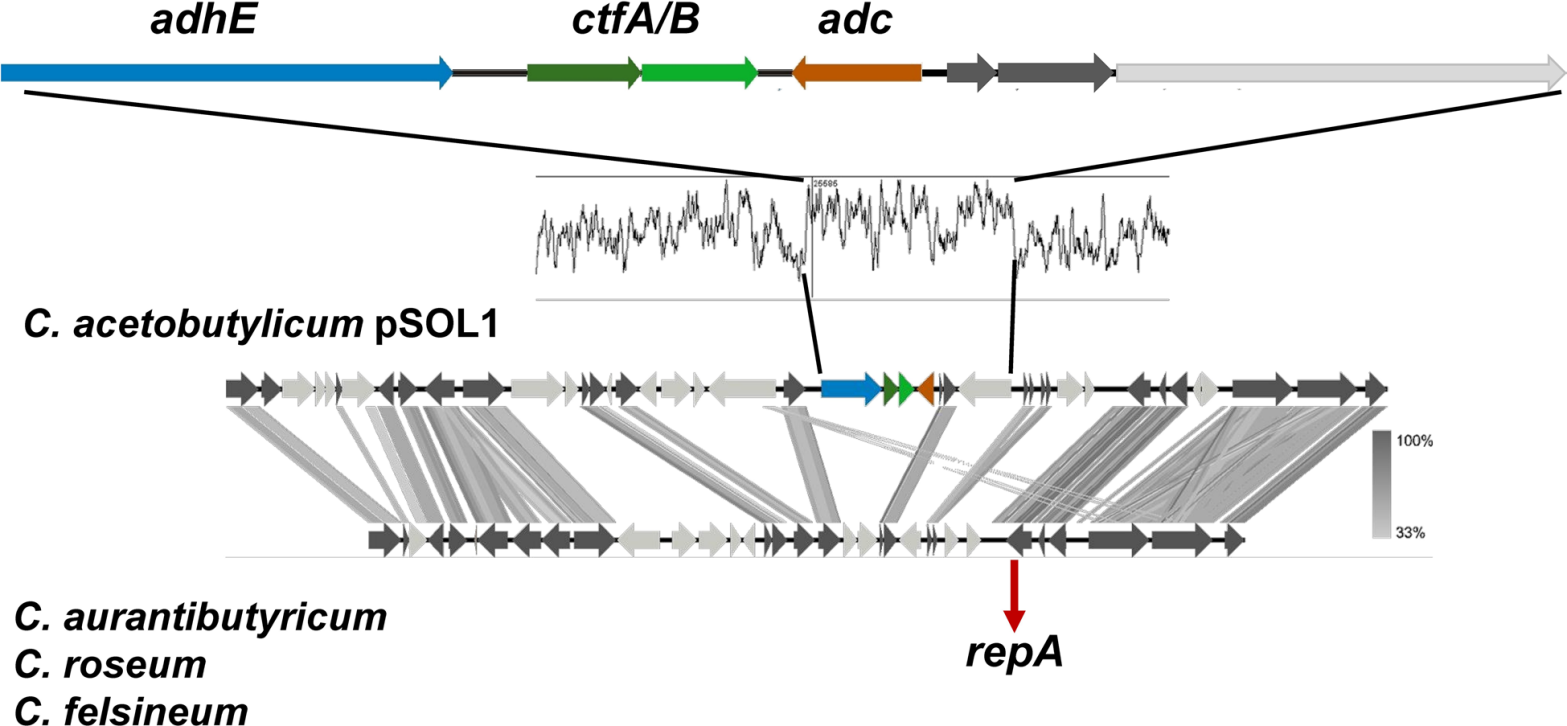
Localization of the *sol* operon: the localization of the *sol* operon in the megaplasmid pSOL1 of *C. acetobutylicum* is compared with the localization in the chromosome of *C. aurantibutyricum*, *C. roseum*, and *C. felsineum*. Visualization was done with Easysfig [21] (tblastx, *E* value cutoff of 1e−10). The GC-content of the *C. acetobutylicum sol* operon is depicted in comparison to the flanking regions

The organization of the genes required for solvent formation fall into two different groups, which correlate well with the two major phylogenetic groupings. Members of the clade *C. acetobutylicum*, *C. aurantibutyricum/C. felsineum/C. roseum*, and *C. pasteurianum* contain a *sol* operon, consisting of *adhE*–*ctfA*–*ctfB* (encoding a bifunctional butyraldehyde/butanol dehydrogenase and the two subunits of CoA transferase), and an adjacent, convergently transcribed, monocistronic *adc* operon (encoding acetoacetate decarboxylase) [20] (Fig. 6). In *C. acetobutylicum* strains, *sol* and *adc* operon reside on the megaplasmid pSOL1, whereas in *C. aurantibutyricum/C. felsineum/C. roseum,* and *C. pasteurianum* these genes are chromosomally located. Nevertheless, *C. aurantibutyricum/C. felsineum/C. roseum* also contain a very similar megaplasmid, but without *sol* and *adc* locus. Interestingly, *sol/adc* operons on the megaplasmid pSOL1 are flanked by inverted repeats, indicative of a mobile element (Fig. 7). The other clade (*C. beijerinckii*, *C. puniceum*, *C. saccharobutylicum*, *C. saccharoperbutylacetonicum*) carries a type II *sol* operon consisting of *ald*–*ctfA*–*ctfB*–*adc* (encoding NADH-dependent aldehyde dehydrogenase, CoA transferase, and acetoacetate decarboxylase) (Fig. 6). Detailed analyses on product formation, including references to respective experimental evidence, and gene clusters required for acidogenesis or solventogenesis, respectively, are presented in Additional file 3: Table S3 and Additional file 4: Table S4.

The availability of the industrial strain collection allowed a direct comparison of sequentially selected strains at the genome level. Much to our surprise, mutations in genes directly required for acidogenesis or solventogenesis were all but absent. The only example was found in *C. beijerinckii* NCP260, a descendant from *C. beijerinckii* BAS/B3/I/124. In NCP 260, a single-nucleotide polymorphism (SNP) was detected in the *ptb* gene, leading to a M122I substitution. When testing the specific activity of phosphotransbutyrylase in this strain, a 54% lower activity was measured compared to the parent (Table 2). A lower capacity for butyrate production leads to higher butanol formation, a trait that is consistent with the past selection of the strain for higher butanol productivity during commercial operation.

**Table 2:** Specific phosphotransbutyrylase activity of different *C. beijerinckii* strains

Strain	Specific PTB activity (U mg^−1^ total protein)	
	8-h growth^a^	27-h growth
*C. beijerinckii* NCIMB 8052	58.2 ± 2.2	67.3 ± 1.2
*C. beijerinckii* BAS/B3/I/124	80.6 ± 4.7	69.1 ± 5.2
*C. beijerinckii* NCP 260	37.1 ± 0.8	29.7 ± 2.9

^a^Values are the average of five independent measurements ± SD

Phosphotransbutyrylase (PTB) activity was determined according to Andersch et al. [57] in CGM cultures of the late strain NCP 260 and the early strain BAS/B3/I/124. The strain NCIMB 8052 was used as a reference

### Substrate utilization

Originally, *C. acetobutylicum* was isolated and grown on starch as the carbon source. Later, strains belonging to the *C. beijerinckii*, *C. puniceum*, *C. saccharobutylicum*, and *C. saccharoperbutylacetonicum* clade were isolated that performed better on molasses-based feedstocks. All strains contained genes for sucrose-specific phosphotransferase systems and sucrose degradation, as well as starch degradation. The only exception with respect to starch degradation is *C. pasteurianum* (Fig. 5). Glycerol transporters are found in all species. Glycolysis and pentose phosphate pathway genes are always present, whereas d-xylose ABC transporter genes are missing in *C. felsineum* and *C. pasteurianum* species. A detailed analysis on the presence or absence of respective genes for substrate degradation, including references to respective experimental evidence, is presented in Additional file 5: Table S5.

### Energy conservation

All 44 ABE strains can synthesize ATP by substrate level phosphorylation during glycolysis (3-phosphoglycerate and pyruvate kinases), acetate (acetate kinase), and butyrate (butyrate kinase) formation, as judged from the genomic repertoire. Also, all strains have genes encoding an F_1_F_O_-ATPase and no genes encoding an energy-conserving hydrogenase (*ech*). However, one major difference is found between the two phylogenetic groups: the *C. beijerinckii*, *C. puniceum*, *C. saccharobutylicum*, and *C. saccharoperbutylacetonicum* clade contains *rnf* genes that encode a protein complex converting reduced ferredoxin to NADH, thereby generating an ion gradient (protons or Na^+^) across the cytoplasmic membrane. This ion gradient can be used for additional ATP synthesis via the ATPase. No members of the *C. acetobutylicum*, *C. aurantibutyricum/C. felsineum/C. roseum*, and *C. pasteurianum* clade possess *rnf* genes.

### Regulators

The presence of several global regulators was checked in all 44 solvent-producing strains. Spo0A is the master regulator of sporulation and also controls the onset of solventogenesis [22, 23], CodY is a pleiotropic regulator involved in degradation of macromolecules, nutrient transport, amino acid and nitrogen metabolism, chemotaxis, solventogenesis, sporulation, and synthesis of antibiotics and branched chain amino acids [24–26]; CcpA is essential for catabolite repression; and Rex controls multiple genes affecting the redox status of the cells [27–30]. All strains contained *spo0A*, *codY*, *ccpA*, and *rex* genes.

### Sporulation proteins and sigma factors

Similar to *Bacillus*, the sporulation process in *Clostridium* is controlled by the orchestrated expression of a series of alternative sigma factors [22, 31, 32]. Homologs of *sigH*, *sigF*, *sigE*, *sigG*, and *sigK* were found in all analyzed strains. The repressor AbrB is involved in the sporulation process. Homologs of *abrB* were identified in all analyzed strains. An analysis on the presence or absence of respective genes for sporulation and sigma factors is presented in Additional file 6: Table S6.

### Quorum sensing

The solvent-producing *Clostridium* species contain multiple peptide-based cell–cell signaling systems homologous to the well-studied *agr* and RNPP-type quorum sensing systems first identified in *Staphylococcus aureus* [33] and *Bacillus* spp. [34], respectively. The *C. saccharoperbutylacetonicum* genomes revealed the presence of five RNPP-type systems in addition to four putative *agr* systems, whereas *C. acetobutylicum* strains were found to only contain a single *agr* locus and eight RNPP-type systems [35]. The different strains of *C. beijerinckii* and *C. saccharobutylicum* contained up to six and three *agr* systems, respectively, but no complete RNPP-type systems. Thus, while physiologically similar and, in some cases, very closely related, these species have evolved rather differently in terms of their ability to communicate.

## Discussion

Since the discovery of biological butanol formation in “*Vibrion butyrique*” (probably a mixed culture) by Louis Pasteur in 1862 [36], numerous anaerobic microorganisms showing the same metabolic property had been isolated and given a multiplicity of different names [5]. Taxonomic principles were applied much later, leading to valid descriptions in 1926 of *C. acetobutylicum* [3] and *C. beijerinckii* [37]. However, even strain deposits in acknowledged culture collections were sometimes spore-contaminated and misclassified, i.e. “*C. acetobutylicum* NCIMB 8052” [38], which was later shown to be a *C. beijerinckii* strain [39, 40]. The designations *C. saccharobutylicum* and *C. saccharoperbutylacetonicum* were introduced with valid descriptions only in 2001 [40]. Here, we present a detailed overview of the ABE-producing clostridia, which clearly fall into two distinct phylogenetic clades. One is formed by *C. acetobutylicum*, *C. aurantibutyricum/C. felsineum*/*C. roseum*, and *C. pasteurianum.* ANIm comparisons show that the differences between *C. aurantibutyricum/C. felsineum*/*C. roseum* are only marginal and do not justify separate species designations. Amended descriptions and a common species name will be required. Conversely, *C. pasteurianum* BC1 does constitute a new species outside of *C. pasteurianum*. The phylogenetic grouping of the *C. acetobutylicum*, *C. aurantibutyricum/C. felsineum*/*C. roseum*, *C. pasteurianum* clade is characterized by (1) the common type I *sol* operon organization (gene order *adhE*–*ctfA*–*ctfB*) and a separate *adc* operon, located adjacent and being transcribed convergently, (2) the absence of *rnf* genes, thus not allowing the generation of an additional ion gradient from reduced ferredoxin, and (3) the presence of a *pdc* gene, encoding pyruvate decarboxylase.

The second clade consists of the most widely used industrial strains (after the switch to invert sugars and molasses as substrate) and includes *C. beijerinckii*, *C. saccharobutylicum*, *C. saccharoperbutylacetonicum*, and *C. puniceum*. Other members are *Clostridium* sp. Maddingley MBC34-24 and the two *Clostridium* species DL_VIII and BL-8, which constitute separate species and will require new descriptions and designations. Misclassified members are *C. pasteurianum* NRRL B-598 and *C. diolis*, which are clearly *C. beijerinckii* species. Also, *C. beijerinckii* HUN142 does not belong to the *C. beijerinckii* group and constitutes a separate species. All members of this second clade possess *rnf* genes and a type II *sol* operon in the gene order *ald*–*ctfA*–*ctfB*–*adc* and they all miss a *pdc* gene.

Solvent formation is mostly restricted to clostridia. Few other bacteria outside of this genus have been reported to be able to produce butanol. However, genome sequences of *Eubacterium limosum* SA11 [41] as well as KIST612 [42] and *Butyribacterium methylotrophicum* [43] reveal that such microorganisms do not possess *sol* operons of either clostridial type. Instead, aldehyde and alcohol dehydrogenase genes are found, whose encoded enzymes catalyze the production of butanol from butyryl-CoA. Within the archaea, only *Hyperthermus butylicus* has been described as a butanol producer [44, 45]. However, this is obviously an experimental flaw as genome sequencing did not reveal respective genes [46] and growth experiments on a variety of substrates never resulted in butanol formation [47]. The presence of a *sol* operon allows cells to couple butyrate conversion and butanol formation and thus to increase unfavorably low pH values to more neutral ones. This mechanism provides an ecological advantage over nutrient competitors (who would die at low pH) allowing sufficient time for spore formation and thus long-time survival. As clostridia are endospore formers, this might be the reason for the evolutionary development of *sol* operons.

It is not obvious why *C. acetobutylicum*, *C. aurantibutyricum/C. felsineum*/*C. roseum*, and *C. pasteurianum* clade members contain a pyruvate decarboxylase (Pdc) but lack an Rnf complex. One possibility involves cofactor recycling. The *pdc* gene in *C. acetobutylicum* is expressed significantly higher during acidogenesis [48]. In contrast to acetone and butanol, ethanol is already formed during the acidogenic stage. Pyruvate is first decarboxylated to acetaldehyde and CO_2_ (by Pdc), and the acetaldehyde is reduced to ethanol (by an alcohol dehydrogenase), requiring only 1 NADH. Conversely, ethanol formation from pyruvate via acetyl-CoA (product of the pyruvate:ferredoxin-oxidoreductase reaction) and acetaldehyde requires 2 NADH. The Rnf complex will produce additional NADH from oxidation of reduced ferredoxin. Thus, it seems that members of the *C. acetobutylicum*, *C. aurantibutyricum/C. felsineum*/*C. roseum*, and *C. pasteurianum* clade cannot reoxidize NADH as easily as the *C. beijerinckii*, *C. puniceum*, *C. saccharobutylicum*, *C. saccharoperbutylacetonicum* clade members and therefore possess a pyruvate decarboxylase and lack an Rnf complex.

Despite the presence of cellobiase- and cellulase-encoding genes, no solventogenic *Clostridium* has ever been reported to utilize cellulose. The genes encoding the putative cellulosome of *C. acetobutylicum* are exclusively transcribed throughout solventogenic growth [48]. Are they translated? If so, what is the function of the proteins during solventogenesis (the medium did not contain cellulose)? These are questions that cannot be answered by a comparative genome analysis and therefore still await experimental elucidation.

The industrial strains within the first clade that were used for the commercial production of solvents from corn are *C. acetobutylicum* DSM1732, EA2018, ATCC 824 and DSM 1731. The industrial strains used for commercial solvent production from molasses include *C. beijerinckii* NCIMB 8052, 4J9, NRRLB-591, and ATCC 35702. A later group of industrial strains successfully used for the commercial production of solvents from molasses are represented by *C. beijerinckii* BAS/B2, BAS/B/1/124, and NCP260. In addition, all of the strains belonging to the *C. saccharobutylicum* cluster and the *C. saccharoperbutylacetonicum* cluster were derived from industrial strains used for solvent production from molasses. With one exception, no key genetic features or characteristics can be identified that would have made these two major groups of successful industrial strains stand out, compared with the other non-industrial strains included in this survey. Only one mutation was identified in genes directly involved in either acid or solvent production (i.e. the *ptb* gene) in all the industrial strains sequenced despite continuous commercial selection for improved solvent production over several decades. However, a similar phenomenon was reported with *Corynebacterium glutamicum*, in which improvement of amino acid production was achieved by mutations unrelated to direct amino acid metabolism [49, 50]. This clearly indicates that bacteria evolved a complex network of metabolic reactions, which influence each other to rebalance concentrations of fermentation products. Instead of focusing on increasing expression of genes for solventogenesis and decreasing expression of genes for acidogenesis, a random mutagenesis approach might be suitable, using, e.g. the newly developed, inducible, mariner-based transposon for *C. acetobutylicum* [51]. In addition, the plethora of genes, stemming from this genome sequencing project, will also allow gene shuffling approaches, leading to more active enzymes.

## Conclusions

Although the ABE fermentation is an established industrial process and the products are both renewable and valuable with respect to the size of both the chemical and biofuel markets (butanol is a superior biofuel to ethanol), the fermentation process has constantly struggled to compete with petrochemical synthesis with respect to feedstock cost and ultimately product pricing. Robust and highly productive strains are required for fermentation at industrial scale, using low-cost feedstocks that do not compete with food. The availability of a multitude of genome sequences from solvent-forming clostridia now supports detailed data mining for less obvious gene mutations and new engineering targets for improved solvent production (e.g. by gene shuffling) with the aim of developing more robust and sustainable fermentation routes for the production of acetone and butanol for chemical and biofuel applications.

## Methods

### Bacterial strains and growth conditions

The strains *C. beijerinckii* BAS/B3/I/124, NCIMB 8052, and NCP260 were maintained as spore suspensions in a modified MS mineral medium [52] and stored at −20 °C. The medium was composed of a basal medium (CaCO_3_ 11.35 mM, KH_2_PO_4_ 8.35 mM, K_2_HPO_4_ 6.52 mM, MgSO_4_ × 7 H_2_O 0.46 mM, (NH_4_)_2_ SO_4_ 19.9 mM, Resazurin 4 µM), a mineral–vitamin solution (NaCl 171 µM, Na_2_MoO_4_ × 2 H_2_O 41.3 µM, CaCl × 2 H_2_O 68 µM, MnSO_4_ × H_2_O 88.7 µM, FeSO_4_ × 7 H_2_O 54 µM, Thiamin–HCl 5.9 µM, *p*-aminobenzoic acid 14.5 µM, Biotin 0.4 µM), and a butyrate solution (0.1 M). 1 ml of the mineral–vitamin solution and 1 ml of the butyrate solution were added to 10 ml glucose (20 g l^−1^) from which 600 µl was mixed to 4.4 ml basal medium. To inoculate cultures, spores were used (pasteurization for 10 min at 80 °C prior cultivation). All other strains were grown in CGM (*Clostridium* growth medium) [53], consisting of 50 g d-glucose × H_2_O, 1 g NaCl, 5 g yeast extract, 0.75 g KH_2_PO_4_, 0.75 g K_2_HPO_4_, 0.71 g MgSO_4_ × 7H_2_O, 2 g (NH_4_)_2_SO_4_, 2.25 g asparagine × H_2_O, 0.01 g MnSO_4_ × H_2_O, 0.01 g FeSO_4_ × 7H_2_O, and 1 mg resazurin per l distilled, anaerobic water. After preparation, the pH of CGM was 6.9. For enzyme assays, cells were grown anaerobically without agitation at 32 °C in 50 ml CGM under anaerobic conditions at 32 °C without agitation.

### Genome sequencing and analysis

Chromosomal DNA was used to prepare shotgun libraries according to the manufacturer’s protocol which were subsequently sequenced (for details see Table 1). Obtained reads were processed and assembled as described in Bengelsdorf et al. [54] (for results see Table 1).

Automatic annotation was performed using the Prokka annotation pipeline [55] and additional analyses were done with the IMG/ER database [56].

Protein sequences from all genomes including the 14 publicly available ones were extracted using cds_extractor.pl v0.6 (https://github.com/aleimba/bac-genomics-scripts) and used for downstream analysis with an in house pipeline (https://github.com/jvollme/PO_2_MLSA) as described in Billerbeck et al. [9]. To calculate the average nucleotide identity of the different genomes, PYANI and the ANIm option was used (https://github.com/widdowquinn/pyani).

### Preparation of cell-free extract and enzyme assays

The *C. beijerinckii* strains BAS/B3/I/124, NCIMB 8052, and NCP260 were grown as described above. Cells were harvested anaerobically after 8 and 27 h by centrifugation at 3214*g* for 10 min at 4 °C, washed twice with 20 ml 0.1 M potassium phosphate buffer pH 7.2 and were stored at −20 °C. Cell pellet was suspended in 1 ml 0.1 M potassium phosphate buffer pH 7.2 and cooled to 0 °C on ice. This mixture was anaerobically transferred to a 2-ml microtube with screw cap containing 0.1-mm glass beads and then cells were disrupted in a RiboLyser™ [Hybaid Ltd., Middlesex (UK)] in five cycles at 6.5 m s^−1^ for 45 s, with breaks of 2 min, during which the extracts were kept on ice. Centrifugation was performed at 38,000*g* for 30 min at 4 °C. Phosphotransbutyrylase (PTB) activity was assayed anaerobically at 37 °C. The enzyme PTB catalyzes the reaction of butyryl-CoA and phosphate to butyryl-phosphate and CoA. The sulfuryl group of the latter was quantified by the absorbance at 405 nm in the presence of DTNB [5,5′-dithiobis-(2-nitrobenzoic acid)]. The activity of PTB in crude extract was measured by monitoring the formation of the reaction product at 405 nm. For activity calculation, the extinction coefficient of 13.6 mM^−1^ cm^−1^ was used. One unit of PTB is defined as the amount of the enzyme that produces 1 µmol of butyryl-CoA per minute under the reaction conditions. The total protein concentration was measured using Pierce BCA Protein Assay Kit (Thermo Scientific). Specific PTB activity was expressed as units (µmol min^−1^) per milligram of protein [U (mg of total protein)^−1^]. PTB activity was determined as described by Andersch et al. [57].

### Accession numbers

These Whole Genome Shotgun projects have been deposited at DDBJ/ENA/GenBank. For details, see Additional file 7: Table S7.

## Additional files

**Additional file 1: Table S1.** ANIm values calculated with PYANI.

**Additional file 2: Table S2.** Plasmids.

**Additional file 3: Table S3.** Products (acids and solvents).

**Additional file 4: Table S4.** Acidogenensis and solventogenesis gene clusters.

**Additional file 5: Table S5.** Substrate degradation.

**Additional file 6: Table S6.** Sporulation proteins and σ-factors.

**Additional file 7: Table S7.** General GenBank features.

## Abbreviations

ABE: acetone–butanol–ethanol
ANI: average nucleotide identity
CGM: *Clostridium* growth medium
CSC: Commercial Solvents Corporation
CS-GB: Commercial Solvents-Great Britain
MSLA: multi-locus sequence analysis
NCP: National Chemical Products
OGs: orthologous groups
SNP: single-nucleotide polymorphism
WW: world war
