# Supplementary material for: Microbial solvent formation revisited by comparative genome analysis
Source: Biotechnol Biofuels. 2017 Mar 9;10:58. doi: 10.1186/s13068-017-0742-z (PMC5343299; doi:10.1186/s13068-017-0742-z)
Supplement: Supplementary file 2 — Additional file 2: Table S2. Plasmids. [file 13068_2017_742_MOESM2_ESM.docx]

**Supplementary Table 2: Plasmids**

| **Plasmid features** | ***C. aceto- butylicum* cluster[1,2,3]** | ***C. beijerinckii* cluster** | ***C. puniceum* DSM2619** | ***C. saccharo- butylicum* cluster** | ***C. saccharo- perbutyl- acetonicum* cluster** | ***Clostridium sp.* cluster ^**^** | ***C. roseum/ C. auranti-butyricum* cluster** | ***C. pasteurianum* cluster** | ***C. felsineum*** |
| --- | --- | --- | --- | --- | --- | --- | --- | --- | --- |
| **Presence** | Yes* | Yes | No | No | Yes | No | Yes | Yes** | Yes |
| **Size** | 11123 – 192000 bp | 1554 - 64987 bp | --- | --- | 2936 – 136188 bp | --- | 31015 - 55559 bp | 53393 bp | 339776 bp |

*: no plasmid present in *Clostridium* *acetobutylicum* GXAS18-1

**: no plasmid present in *Clostridium* *pasteurianum* CP1 and DSM525

1. Fischer RJ, Helms J, Dürre P. Cloning, sequencing, and molecular analysis of the sol operon of *Clostridium acetobutylicum*, a chromosomal

locus involved in solventogenesis. J Bacteriol 1993;175:6959-69.

1. Cornillot E, Nair RV, Papoutsakis ET, Soucaille P. The genes for butanol and acetone formation in *Clostridium acetobutylicum* ATCC 824 reside on a large plasmid whose loss leads to degeneration of the strain. J Bacteriol 1997;179:5442-7.
2. Bao G, Wang R, Zhu Y, Dong H, Mao S, Zhang Y, Chen Z, Li Y, Ma Y. Complete genome sequence of *Clostridium acetobutylicum* DSM 1731, a solvent-producing strain with multireplicon genome architecture. J Bacteriol 2011;193:5007-8.
