# Supplementary material for: Microbial solvent formation revisited by comparative genome analysis
Source: Biotechnol Biofuels. 2017 Mar 9;10:58. doi: 10.1186/s13068-017-0742-z (PMC5343299; doi:10.1186/s13068-017-0742-z)
Supplement: Supplementary file 3 — Additional file 3: Table S3. Products (acids and solvents). [file 13068_2017_742_MOESM3_ESM.docx]

**Supplementary Table 3: Products (acids and solvents)**

| **Product** | **Parameter** | ***C. aceto- butylicum* cluster[5]** | ***C. beijerinckii* cluster[10]** | ***C. puniceum* DSM2619[1]** | ***C. saccharo- butylicum* cluster[13]** | ***C. saccharo- perbutyl- acetonicum* cluster[14]** | ***Clostridium sp.* cluster ^**^** | ***C. roseum/ C. auranti-butyricum* cluster[2, 3]** | ***C. pasteurianum* cluster** [**4, 15]** | ***C.*** *felsineum*[4] |
| --- | --- | --- | --- | --- | --- | --- | --- | --- | --- | --- |
| **Acetate** | **Full pathway in genome** | Yes | Yes | Yes | Yes | Yes | Yes | Yes | Yes | Yes |
|  | **Experimental evidence** | Yes | Yes | Yes | Yes | Yes | No | Yes | Yes | No |
| **Butyrate** | **Full pathway in genome** | Yes | Yes | Yes | Yes | Yes | Yes | Yes | Yes | Yes |
|  | **Experimental evidence** | Yes | Yes | Yes | Yes | Yes | No | Yes | Yes | No |
| **Lactate** | **Full pathway in genome** | Yes | Yes | Yes | Yes | Yes | Yes | Yes | Yes | Yes |
|  | **Experimental evidence** | Yes[6] | Yes[11] | No | No | No | No | Yes | Yes | Yes |
| **Acetone** | **Full pathway in genome** | Yes^#^ | Yes | Yes | Yes | Yes | (Yes)^*^ | Yes | Yes | Yes |
|  | **Experimental evidence** | Yes | Yes | Yes | Yes | Yes | No | Yes | Yes | Yes[2,3] |
| **Butanol** | **Full pathway in genome** | Yes^#^ | Yes | Yes | Yes | Yes | Yes | Yes | Yes | Yes |
|  | **Experimental evidence** | Yes | Yes | Yes | Yes | Yes | No | Yes | Yes | Yes[2,3] |
| **Ethanol** | **Full pathway in genome** | Yes^#^ | Yes | Yes | Yes | Yes | Yes | Yes | Yes | Yes |
|  | **Experimental evidence** | Yes | Yes | Yes | Yes | Yes | No | Yes | Yes | Yes[2,3] |
| **Acetoin** | **Full pathway in genome** | Yes | Yes | No | No | No | No | Yes | No | Yes |
|  | **Experimental evidence** | Yes[7] | No[9] | No | No | No | No | No | No | No |
| **2,3-Butanediol** | **Full pathway in genome** | No | Yes | No | No | No | No | No | No | No |
|  | **Experimental evidence** | No[7] | No | No | No | No | No | No | No | No |
| **1,3-Propanediol** | **Full pathway in genome** | No | Yes | No | No | No | No | No | Yes | No |
|  | **Experimental evidence** | No[8] | Yes[12] | No | No | No | No | No | Yes | No |
| **Isopropanol** | **Full pathway in genome** | No | Yes | Yes | No | No | Yes^**^ | Yes^***^ | No | No |
|  | **Experimental evidence** | No[9] | Yes[9] | No | No | No | No | No | No | No |

*: *ctfA/B* not in *Clostridium sp.* Maddingley

**: Dehydrogenase gene not in *Clostridium sp.* Maddingley

***: Dehydrogenase gene not in *C. aurantibutyricum* DSM793

^#^: *sol* and *adc* not in *Clostridium acetobutylicum* GXAS18-1

1. Holt RA, Cairns AJ, Morris JG. Production of butanol by *Clostridium puniceum* in batch and continuous culture. Appl Microbiol Biotechnol 1988;27:319-24.
2. McCoy E and McClung LS. Studies on anaerobic bacteria. IV. The nature and systematic position of a new chromogenic *Clostridium*. Arch Mikrobiol 1935;6:230-8.
3. Hellinger, E. *Clostridium aurantibutyricum* (n.sp.): A pink butyric acid *Clostridium*. J Gen Microbiol 1947;1:203-10.
4. Masset J, Calusinska M, Hamilton C, Hiligsmann S, Joris B, Wilmotte A, Thonart P. Fermentative hydrogen production from glucose and starch using pure strains and artificial co-cultures of *Clostridium* spp. Biotechnol Biofuels 2012;5:35(1-15).
5. Fond O, Matta-Ammouri G, Petitdemange H, Engasser JM. The role of acids on the production of acetone and butanol by *Clostridium acetobutylicum*. Appl Microbiol Biotechnol 1985;22:195-200.
6. Medkor N, Zerdani I, Sattar S, El Kanouni A, Petitdemange H. Isolation of *Clostridium acetobutylicum* ATCC824 mutants using propionic and isovaleric acid halogen analogues as suicide substrates. Int J Microbiol Res 2010;1:22-5.
7. Siemerink MAJ, Kuit W, López Contreras AM, Eggink G, van der Oost J, Kengen SWM. D-2,3-butanediol production due to heterologous expression of an acetoin reductase in *Clostridium acetobutylicum*. Appl Environ Microbiol 2011;77:2582–8.
8. González-Pajuelo M, Meynial-Salles I, Mendes F, Andrade JC, Vasconcelos I, Soucaille P. Metabolic engineering of *Clostridium acetobutylicum* for the industrial production of 1,3-propanediol from glycerol. Metab Eng 2005;7:329-36.
9. Collas F, Kuit W, Clément B, Marchal R, López-Contreras AM, Monot F. Simultaneous production of isopropanol, butanol, ethanol and 2,3-butanediol by *Clostridium acetobutylicum* ATCC 824 engineered strains. AMB Express 2012;2:45(1-10).
10. Chen CK, Blaschek HP. Effect of acetate on molecular and physiological aspects of *Clostridium beijerinckii* NCIMB 8052 solvent production and strain degeneration. Appl Environ Microbiol 1999;65:499-505.
11. Wang Y, Li X, Milne CB, Janssen H, Lin W, Phan G, Hu H, Jin YS, Price ND, Blaschek HP. Development of a gene knockout system using mobile group II introns (Targetron) and genetic disruption of acid production pathways in *Clostridium beijerinckii*. Appl Environ Microbiol 2013;79:5853-63.
12. Otte B, Grunwaldt E, Mahmoud O, Jennewein S. Genome shuffling in *Clostridium diolis* DSM 15410 for improved 1,3-propanediol production. Appl Environ Microbiol 2009;75:7610-6.
13. Ni Y, Wang Y, Sun Z. Butanol production from cane molasses by *Clostridium saccharobutylicum* DSM 13864: batch and semicontinuous fermentation. Appl Biochem Biotechnol 2012;166:1896-907.
14. Thang VH, Kanda K, Kobayashi G. Production of acetone-butanol-ethanol (ABE) in direct fermentation of cassava by *Clostridium saccharoperbutylacetonicum* N1-4. Appl Biochem Biotechnol 2010;161:157-70.
15. Harris J, Mulder R, Kell DB, Walter RP, Morris JG. Solvent production by *Clostridium pasteurianum* in media of high sugar content. Biotechnol Lett 1986;8:889–92.
