# Supplementary material for: Microbial solvent formation revisited by comparative genome analysis
Source: Biotechnol Biofuels. 2017 Mar 9;10:58. doi: 10.1186/s13068-017-0742-z (PMC5343299; doi:10.1186/s13068-017-0742-z)
Supplement: Supplementary file 4 — Additional file 4: Table S4. Acidogenensis and solventogenesis gene clusters. [file 13068_2017_742_MOESM4_ESM.docx]

**Supplementary Table 4: Acidogenensis and solventogenesis gene clusters**

| **Gene cluster** | **Operon** | ***C. aceto- butylicum* cluster[1,2]** | ***C. beijerinckii* cluster[1,2]** | ***C. puniceum* DSM2619** | ***C. saccharo- butylicum* cluster[1,2]** | ***C. saccharo- perbutyl- acetonicum* cluster[1,2]** | ***Clostridium sp.* cluster** | ***C. roseum/ C. auranti-butyricum* cluster** | ***C. pasteurianum* cluster** | ***C. felsineum*** |
| --- | --- | --- | --- | --- | --- | --- | --- | --- | --- | --- |
| ***sol-adc*** | ***sol*** | Megaplasmid^#^  *adhE*, *ctfA*, *ctfB* | Chromosome  *ald*, *ctfA*, *ctfB*, *adc* | Chromosome  *ald*, *ctfA*, *ctfB*, *adc* | Chromosome  *aldA*, *ctfA*, *ctfB*, *adcA* | Chromosome  *bld*, *ctfA*, *ctfB*, *adc* | Chromosome  *ald*^*^, *ctfA*, *ctfB*, | Chromosom  *adhE*, *ctfA*, *ctfB*, | Chromosome  *adhE*, *ctfA*, *ctfB*, | Chromosome  *adhE*, *ctfA*, *ctfB*, |
|  | ***adc*** | Megaplasmid  *adc* | --- | --- | --- | --- | Chromosome  *adc* | Chromosom  *adc* | Chromosome  *adc* | Chromosome  *adc* |
| ***bcs-adhA*** | ***bcs*** | Chromosome^#^  *crt*, *bcd*, *etfB*, *etfA*, *hbd* | Chromosome  *crt*, *bcd*, *etfB*, *etfA*, *hbd* | Chromosome  *crt*, *bcd*, *etfB*, *etfA*, *hbd* | Chromosome  *crtA*, *bcdA*, *etfB*, *etfA*, *hbdA* | Chromosome  *crt*, *bcd*, *etfB*, *etfA*, *hbd* | Chromosome  *crt*, *bcd*, *etfB*, *etfA*, *hbd* | Chromosome  *crt*, *bcd*, *etfB*, *etfA*, *hbd* | Chromosome^xxx^  *crt*, *bcd*, *etfB*, *etfA*, *hbd* | Chromosome  *crt*, *bcd*, *etfB*, *etfA*, *hbd* |
|  | ***adhA*** | --- | --- | Chromosome  *adh* | Chromosome  *adhA* | Chromosome  *adh* | Chromosome  *adh* | --- | --- | --- |

^*^: *Clostridium sp.* DL-VIII has no *ald* (a propionaldehyde dehydrogenase is present in the *sol* operon)

^#^: *sol* and *adc* not in *Clostridium* *acetobutylicum* GXAS18-1

1. Berezina OV, Brandt A, Yarotsky S, Schwarz WH, Zverlov VV. Isolation of a new butanol-producing *Clostridium* strain: high level of hemicellulosic activity and structure of solventogenesis genes of a new *Clostridium saccharobutylicum* isolate. Syst Appl Microbiol 2009;32:449-59.
2. Fischer RJ, Helms J, Dürre P. Cloning, sequencing, and molecular analysis of the *sol* operon of *Clostridium acetobutylicum*, a chromosomal locus involved in solventogenesis. J Bacteriol 1993;175:6959-69.
