# Supplementary material for: Microbial solvent formation revisited by comparative genome analysis
Source: Biotechnol Biofuels. 2017 Mar 9;10:58. doi: 10.1186/s13068-017-0742-z (PMC5343299; doi:10.1186/s13068-017-0742-z)
Supplement: Supplementary file 5 — Additional file 5: Table S5. Substrate degradation. [file 13068_2017_742_MOESM5_ESM.docx]

**Supplementary Table 5: Substrate degradation**

| **Substrate** | **Parameter** | ***C. aceto- butylicum* cluster[1]** | ***C. beijerinckii* cluster** | ***C. puniceum* DSM2619[7, 8]** | ***C. saccharo- butylicum* cluster[9]** | ***C. saccharo- perbutyl- acetonicum* cluster[9]** | ***Clostridium sp.* ^**^ cluster** | ***C. roseum/ C. auranti-butyricum* cluster[**11,12] | ***C. pasteurianum* cluster[**13, 14] | ***C.* felsineum[**14] |
| --- | --- | --- | --- | --- | --- | --- | --- | --- | --- | --- |
| **Cellobiose** | **Full pathway in genome** | Yes | No | Yes | Yes | Yes | No | Yes | No | No |
|  | **PTS transporter** | Yes | Yes | Yes | Yes | Yes | Yes | Yes | No | No |
|  | **Experimental evidence** | No[2] | No[3] | No | No[10] | No[10] | No | No | No | No |
| **Sucrose** | **Full pathway in genome** | Yes | Yes | Yes | Yes | Yes | Yes | Yes | Yes | Yes |
|  | **PTS transporter** | Yes | Yes | Yes | Yes | Yes | Yes | Yes | Yes | Yes |
|  | **Experimental evidence** | Yes | Yes[4] | Yes | Yes | Yes | No | Yes | No | No |
| **Starch** | **Full pathway in genome** | Yes | Yes | Yes | Yes | Yes | Yes** | Yes | No | Yes |
|  | **PTS transporter** | Yes | Yes | Yes | Yes | Yes | No | Yes | No | Yes |
|  | **Experimental evidence** | Yes | Yes[5] | Yes | Yes | Yes | No | Yes | No | Yes |
| **Mannose** | **Full pathway in genome** | Yes | Yes | Yes | Yes | Yes | Yes | Yes | Yes | Yes |
|  | **PTS transporter** | Yes | Yes | Yes | Yes | Yes | Yes | Yes | Yes | Yes |
|  | **Experimental evidence** | Yes | Yes[6] | Yes | Yes | Yes | No | Yes | No | No |
| **Xylose** | **Full pathway in genome** | Yes | Yes | Yes | Yes | Yes | Yes | Yes | No | No |
|  | **Symporter/ABC transporter** | Yes | Yes | Yes | Yes | Yes | Yes | Yes | No | Yes |
|  | **Experimental evidence** | Yes | Yes[3] | Yes | Yes | Yes | No | Yes | No | No |
| **Glycerol** | **Full pathway in genome** | Yes | Yes | Yes | Yes | Yes | Yes | No | Yes | Yes |
|  | **Glycerol uptake facilitator** | Yes | Yes | Yes | Yes | Yes | Yes | Yes | Yes | Yes |
|  | **Experimental evidence** | Yes | Yes[3] | Yes | Yes | Yes | No | No | Yes[13] | No |

*: *Clostridium* sp. BL-8, *Clostridium* sp. DL-VIII, *Clostridium* sp. Maddingley

**: *Clostridium* sp. BL-8 cannot utilize starch.

1. Servinsky MD, Kiel JT, Dupuy NF, Sund CJ. Transcriptional analysis of differential carbohydrate utilization by *Clostridium acetobutylicum*. Microbiology 2010;156:3478–91.
2. Sabathé F, Bélaïch A, Soucaille P. Characterization of the cellulolytic complex (cellulosome) of *Clostridium acetobutylicum*. FEMS Microbiol Lett 2002;217:15-22.
3. Watson JE. Pentose sugar utilisation in *Clostridium beijerinckii* NCIMB 8052 for biobutanol production: genetic and physiological studies. (Ph.D. thesis, Edinburgh Napier University, 2012).
4. Tangney M, Rousse C, Yazdanian M, Mitchell WJ. Note: Sucrose transport and metabolism in *Clostridium beijerinckii* NCIMB 8052. J Appl Microbiol 1998;84:914–9.
5. Formanek J, Mackie R, Blaschek HP. Enhanced butanol production by *Clostridium beijerinckii* BA101 grown in semidefined P2 medium containing 6 percent maltodextrin or glucose. Appl Environ Microbiol 1997;63:2306–10.
6. Qureshi N, Saha BC, Cotta MA. Butanol production from wheat straw hydrolysate using *Clostridium beijerinckii*. Bioprocess Biosyst Eng 2007;30:419–27.
7. Lund BM, Brocklehurst TF, Wyatt GM. Characterization of strains of *Clostidium puniceum* sp.nov., a pink-pigmented, pectolytic bacterium. J Gen Microbiol 1981;122:17-26.
8. Holt RA, Cairns AJ, Morris JG. Production of butanol by *Clostridium puniceum* in batch and continuous culture. Appl Microbiol Biotechnol 1988;27:319-24.
9. Keis S, Shaheen R, Jones DT. Emended descriptions of *Clostridium acetobutylicum* and *Clostridium beijerinckii*, and descriptions of *Clostridium saccharoperbutylacetonicum* sp. nov. and *Clostridium saccharobutylicum* sp. nov. Int J Syst Evol Microbiol 2001;51:2095–103.
10. Berezina OV, Zakharova NV, Yarotskya CV, and Zverlov VV. Microbial producers of butanol. Appl Biochem Microbiol 2012;48:625–38.
11. McCoy E and McClung LS. Studies on anaerobic bacteria. IV. The nature and systematic position of a new chromogenic *Clostridium*. Arch Mikrobiol 1935;6:230-8.
12. Hellinger, E. *Clostridium aurantibutyricum* (n.sp.): A pink butyric acid *Clostridium*. J Gen Microbiol 1947;1:203-10.
13. Taconi KA, Venkataramanan KP, Johnson DT. Growth and solvent production by *Clostridium pasteurianum* ATCC®6013™ utilizing biodiesel-derived crude glycerol as the sole carbon source. Environ Prog 2009;28:100-10.
14. Masset J, Calusinska M, Hamilton C, Hiligsmann S, Joris B, Wilmotte A, Thonart P. Fermentative hydrogen production from glucose and starch using pure strains and artificial co-cultures of *Clostridium* spp. Biotechnol Biofuels 2012;5:35(1-15).
