# Supplementary material for: Microbial solvent formation revisited by comparative genome analysis
Source: Biotechnol Biofuels. 2017 Mar 9;10:58. doi: 10.1186/s13068-017-0742-z (PMC5343299; doi:10.1186/s13068-017-0742-z)
Supplement: Supplementary file 6 — Additional file 6: Table S6. Sporulation proteins and σ-factors. [file 13068_2017_742_MOESM6_ESM.docx]

**Supplementary Table 6: Sporulation proteins and σ-factors**

| **Function** | **Parameter** | ***C. aceto- butylicum* cluster** | ***C. beijerinckii* cluster** | ***C. puniceum* DSM2619** | ***C. saccharo- butylicum* cluster** | ***C. saccharo- perbutyl- acetonicum* cluster** | ***Clostridium sp.* cluster ^**^** | ***C. roseum/ C. auranti-butyricum* cluster** | ***C. pasteurianum* cluster** | ***C. felsineum*** |
| --- | --- | --- | --- | --- | --- | --- | --- | --- | --- | --- |
| **Orphan histidine kinases** | **Presence** | Yes | No | No | No | No | No* | Yes | No | Yes |
|  | **Evidence of functionality** | Yes | No | No | No | No | No | No | No | Yes |
| **Sigma factors** | **Presence** | Yes | Yes** | Yes** | Yes** | Yes** | Yes** | Yes** | Yes** | Yes** |
|  | **Evidence of functionality** | Yes | Yes | Yes | Yes | No | No | No | No | No |
| **Sporulation repressors** | **Presence** | Yes | Yes | Yes | Yes | Yes | Yes | Yes | Yes | Yes |
|  | **Evidence of functionality** | Yes | No | No | No | No | No | No | No | No |
| **Sporulation proteins** | **Presence** | Yes | Yes*** | Yes*** | Yes*** | Yes*** | Yes*** | Yes | Yes | Yes |
|  | **Evidence of functionality** | Yes | Yes | Yes | Yes | No | No | No | No | No |

*: *Clostridium sp.* BL-8 has no orphan histidine kinase

**: no homologous sigA is present

***: some genes for typical sporulation proteins[1] are missing

Clostridial sporulation proteins and sigma factors were described by Al-Hinai et al. [1].

1. Al-Hinai MA, Jones SW, Papoutsakis ET. The *Clostridium* sporulation programs: diversity and preservation of endospore differentiation. Microbiol Mol Biol Rev 2015;79:19-37.
