# Supplementary material for: Microbial solvent formation revisited by comparative genome analysis
Source: Biotechnol Biofuels. 2017 Mar 9;10:58. doi: 10.1186/s13068-017-0742-z (PMC5343299; doi:10.1186/s13068-017-0742-z)
Supplement: Supplementary file 7 — Additional file 7: Table S7. General GenBank features. [file 13068_2017_742_MOESM7_ESM.docx]

**Supplementary Table 7: General GenBank features**

| **Organism** | **LocusTag** | **BioProject** | **BioSample** | **status** | **accession number** |
| --- | --- | --- | --- | --- | --- |
| *Clostridium acetobutylicum* DSM 1732 | CLABU | PRJNA322795 | SAMN05170516 | d | LZYY00000000 |
| *Clostridium acetobutylicum* NCCB 24020 | CLACE | PRJNA322794 | SAMN05170515 | d | LZYX00000000 |
| *Clostridium aurantibutyricum* DSM 793T | CLAUR | PRJNA322793 | SAMN05170514 | d | LZYW00000000 |
| *Clostridium beijerinckii* 4J9 | CLOSB | PRJNA322808 | SAMN05170529 | d | LZZG00000000 |
| *Clostridium beijerinckii* ATCC 39058 | CBEIJ | PRJNA322807 | SAMN05170528 | d | LZZF00000000 |
| *Clostridium beijerinckii* BAS/B2 | CLBEJ | PRJNA322804 | SAMN05170525 | d | LZZD00000000 |
| *Clostridium beijerinckii* BAS/B3/I/124 | CLBIJ | PRJNA322805 | SAMN05170526 | f | CP016090 |
| *Clostridium beijerinckii* DSM 53 | CLBCK | PRJNA322810 | SAMN05170531 | d | LZZI00000000 |
| *Clostridium beijerinckii* DSM 791T | CLBEI | PRJNA322803 | SAMN05170524 | d | LZZC00000000 |
| *Clostridium beijerinckii* 59B [1] | LF65 | PRJNA260606 | SAMN03024437 | f | CP010086 |
| *Clostridium beijerinckii* NCP 260 | CLOBJ | PRJNA322806 | SAMN05170527 | d | LZZE00000000 |
| *Clostridium beijerinckii* NRRL B-528 | CLBEIC | PRJNA322812 | SAMN05170533 | d | LZZK00000000 |
| *Clostridium beijerinckii* NRRL B-591 | CLBKI | PRJNA322809 | SAMN05170530 | d | LZZH00000000 |
| *Clostridium beijerinckii* NRRL B-593 | CLOBI | PRJNA322811 | SAMN05170532 | d | LZZJ00000000 |
| *Clostridium beijerinckii* NRRL B-596 | CLOBE | PRJNA322802 | SAMN05170523 | d | LZZB00000000 |
| *Clostridium felsineum* DSM 794T | CLFEL | PRJNA322790 | SAMN05170511 | d | LZYT00000000 |
| *Clostridium pasteurianum* DSM 525T[2] | CLPA | PRJNA258199 | SAMN02990135 | f | CP009268 |
| *Clostridium puniceum* DSM 2619T | CLPUN | PRJNA322815 | SAMN05170536 | d | LZZM00000000 |
| *Clostridium roseum* DSM 6424 | CLROS | PRJNA322791 | SAMN05170512 | d | LZYU00000000 |
| *Clostridium roseum* DSM 7320T | CROST | PRJNA322792 | SAMN05170513 | d | LZYV00000000 |
| *Clostridium saccharobutylicum* BAS/B3/SW/136 | CSACC | PRJNA322798 | SAMN05170519 | f | CP016089 |
| *Clostridium saccharobutylicum* DSM 13864T[3] | CLSA | PRJNA217481 | SAMN02603278 | f | CP006721 |
| *Clostridium saccharobutylicum* L1-8 | CLOSAC | PRJNA322819 | SAMN05170517 | d | LZYZ00000000 |
| *Clostridium saccharobutylicum* NCP 162 | CLSAB | PRJNA322801 | SAMN05170522 | f | LZZA00000000 |
| *Clostridium saccharobutylicum* NCP 195 | CLOSACC | PRJNA322820 | SAMN05170518 | f | CP016092 |
| *Clostridium saccharobutylicum* NCP 200 | CLOSC | PRJNA322799 | SAMN05170520 | f | CP016086 |
| *Clostridium saccharobutylicum* NCP 258 | CLOBY | PRJNA322800 | SAMN05170521 | f | CP016091 |
| *Clostridium saccharoperbutylacetonicum* N1-4 (HMT)T[4] | CSPA | PRJNA60605 | SAMN02603265 | f | CP004121-CP004122 |
| *Clostridium saccharoperbutylacetonicum* N1-504 | CLSAP | PRJNA322814 | SAMN05170535 | f | CP016087-CP016088 |
| Clostridium sp. BL-8 | CLOBL | PRJNA322813 | SAMN05170534 | d | LZZL00000000 |
|  |  |  |  |  |  |
|  |  |  |  |  |  |
| f, finished |  |  |  |  |  |
| d, draft |  |  |  |  |  |

1. Little GT, Winzer K, Minton NP. Genome sequence of the solvent-producing *Clostridium beijerinckii* strain 59B, isolated from Staffordshire garden soil. Genome Announc 2015;3(2):e00108-15.
2. Poehlein A, Grosse-Honebrink A, Zhang Y, Minton NP, Daniel R. Complete genome sequence of the nitrogen-fixing and solvent-producing *Clostridium pasteurianum* DSM 525. Genome Announc 2015;3(1):e01591-14.
3. Poehlein A, Hartwich K, Krabben P, Ehrenreich A, Liebl W, Dürre P, Gottschalk G, Daniel R. Complete genome sequence of the solvent producer *Clostridium saccharobutylicum* NCP262 (DSM 13864). Genome Announc 2013;1(6):e00997-13.
4. Poehlein A, Krabben P, Dürre P, Daniel R. Complete genome sequence of the solvent producer *Clostridium saccharoperbutylacetonicum* strain DSM 14923. Genome Announc 2014;2(5):e01056-14.
